# Ovarian tumor domain-containing protein 1 deficiency attenuates metabolic dysfunction-associated steatotic liver disease by promoting the ubiquitination of HSP90α in hepatocytes

**DOI:** 10.1186/s43556-026-00595-1

**Published:** 2026-09-20

**Authors:** Mei-Qin Huang, Si-Yi Wei, Meng-Zhe Li, Tian-Yu Chen, Shu-Qin Ou, Meng Gao, Feng Shi, Tao Zhang, Ying-Lan Liu, Meng-Zhuo Qin, Run-Lei Du, Qi Zhang, Bai-Qi Wang, Xiao-Dong Zhang

**Affiliations:** 1 Hunan Research Center of the Basic Discipline for Mitochondrial Homeostasis & MOE Key Lab of Rare Pediatric Diseases, School of Basic Medical Sciences & The Second Affiliated Hospital, Hengyang Medical School, University of South China, Hengyang, 421001 China; 2 The First Affiliated Hospital, Hengyang Medical School, University of South China, Hengyang, China; 3 Guangxi Key Laboratory of Artificial Intelligence for Genetic Diseases of Long-dwelling Nationalities, Youjiang Medical University for Nationalities, Baise, Guangxi 533000 China; 4 The Second Affiliated Hospital, Hengyang Medical School, University of South China, Hengyang, China; 5 National “111” Center for Cellular Regulation and Molecular Pharmaceutics, Key Laboratory of Fermentation Engineering (Ministry of Education), Cooperative Innovation Center of Industrial Fermentation (Ministry of Education & Hubei Province), Hubei Key Laboratory of Industrial Microbiology, Hubei University of Technology, Wuhan, 430068 China

**Keywords:** OTUD1, MASLD, HSP90α, Lipid metabolism, Inflammation, Deubiquitination

## Abstract

Metabolic dysfunction-associated steatotic liver disease (MASLD) is the most prevalent chronic liver disease worldwide, yet approved pharmacotherapies remain limited. The ovarian tumor domain-containing protein 1 (OTUD1) has been implicated in metabolic regulation, but its role in MASLD remains unclear. OTUD1 expression was significantly elevated in liver samples from patients with MASLD and in high-fat diet (HFD)-fed mice, a finding further supported by two independent Gene Expression Omnibus (GEO) cohorts (GSE24807 and GSE126848). Studies using systemic *Otud1*-knockout mice showed that *Otud1* deficiency markedly alleviated insulin resistance and inflammation under HFD and high-fat, methionine-restricted, choline-deficient (HFMCD) diet conditions. To assess the hepatocyte-specific and therapeutic relevance of OTUD1, *Otud1* was silenced in hepatocytes using AAV8-TBG-sh*Otud1*, which significantly ameliorated HFMCD-induced steatohepatitis. Mechanistically, integrated interactome analyses identified heat shock protein 90α (HSP90α) as a direct substrate of OTUD1. OTUD1 bound to HSP90α and removed its K48-linked polyubiquitin chains at K283, thereby preventing its proteasomal degradation and enhancing its stability. This effect was required for OTUD1-driven lipid accumulation in hepatocytes, as the catalytically inactive OTUD1-C320S mutant failed to stabilize HSP90α or promote lipid deposition. Transcriptomic profiling further revealed that *OTUD1* overexpression activated lipid metabolism and inflammatory pathways in OA/PA-treated LO2 cells. Together, these findings identify OTUD1 as a previously unrecognized driver of MASLD progression through the OTUD1–HSP90α axis and suggest that OTUD1 may serve as a potential therapeutic target for metabolic liver disease.

**Supplementary Information:**

The online version contains supplementary material available at https://doi.org/10.1186/s43556-026-00595-1.

## Introduction

Metabolic dysfunction-associated steatotic liver disease (MASLD) ranges from simple steatosis to metabolic dysfunction-associated steatohepatitis (MASH), which can progress to fibrosis, cirrhosis, and hepatocellular carcinoma [1–5]. As a highly prevalent condition affecting approximately 38% of adults worldwide, MASLD has become a major public health burden [6–9]. However, the currently approved pharmacotherapies for MASLD are very limited because of the complex pathophysiology and heterogeneity of MASLD [10, 11], highlighting the urgent need to identify new medical therapies for MASLD. Investigations into the pathophysiology and regulatory mechanisms of MASLD will facilitate drug development.

The ubiquitin–proteasome system plays a pivotal role in regulating protein stability and turnover [12, 13]. Targeting the ubiquitination or deubiquitination of critical disease regulators is a highly promising treatment strategy [3, 14–17]. Ubiquitin (Ub), a conserved protein, is covalently conjugated to lysine residues on cellular proteins to regulate proteasome degradation, signal transduction, and gene transcription. This reversible modification is counteracted by deubiquitinating enzymes (DUBs), which remove Ub to stabilize proteins. To date, approximately 100 DUBs have been implicated in health and disease [12, 13, 18–20]. Specifically, DUBs play divergent roles in hepatic metabolic regulation: enzymes such as USP14, RPN11, and USP24 exacerbate steatosis, lipogenesis, and fibrosis [14, 15, 21], whereas others, including USP10 and USP29, confer protection against disease progression [22, 23]. Owing to their well-defined catalytic pockets, DUBs represent highly attractive and pharmacologically tractable drug targets [24–27]. However, the contribution of DUBs to MASLD pathogenesis remains insufficiently understood, and whether specific DUBs may serve as therapeutic targets in MASLD/MASH is largely unexplored.

To identify DUBs potentially involved in MASLD, a gene-expression-based screen was performed in steatotic livers, which identified OTUD1 as a markedly upregulated candidate. The ovarian tumor protease (OTU) family orchestrates signaling pathways that govern inflammation, metabolism, and DNA replication [28]. As an OTU member, OTUD1 specifically hydrolyzes ubiquitin chains conjugated to substrate proteins, including pivotal regulators such as STAT3, p53, and YAP [29–32]. Although OTUD1 plays key roles in many pathological conditions [31, 33–37], its expression pattern, biological function, and downstream substrates in MASLD have not been defined.

Heat shock protein 90α (HSP90α) is a crucial member of the HSP90 family of molecular chaperones, which are proteins essential for cellular homeostasis, particularly under conditions of stress [38]. Its primary function is to facilitate the proper folding, stabilization, activation, and maturation of a diverse set of client proteins. Unlike its constitutive counterpart HSP90β, HSP90α is highly inducible, meaning that its expression is significantly upregulated in response to cellular stressors such as heat, hypoxia, and pH changes. Beyond this canonical role, emerging evidence indicates that HSP90α plays pivotal roles in hepatic lipid metabolism and inflammation [14, 39, 40]. In lipid synthesis, HSP90α promotes lipogenesis by stabilizing fatty acid synthase (FASN) and enhancing FASN transcription via the LXRα pathway, thereby contributing to hepatic lipid accumulation in hepatocellular carcinoma [39]. Clinical studies have confirmed that elevated circulating HSP90α levels in overweight/obese children are closely correlated with MASLD susceptibility [41]. In addition to lipid regulation, HSP90α modulates inflammatory cascades and exacerbates tissue inflammation in metabolic disorders [40]. As a circulating biomarker and therapeutic target, HSP90α links metabolic dysregulation to fatty liver disease progression. Its dual involvement in lipogenic pathway activation and inflammatory response amplification highlights its centrality in conditions ranging from pediatric MASLD to hepatocellular carcinoma, offering promising avenues for targeted interventions in metabolic syndrome-related disorders.

In this study, the role of OTUD1 in MASLD and the molecular mechanism underlying its action were investigated. It was hypothesized that OTUD1 promotes MASLD progression through its deubiquitinating activity, and it was further proposed that this effect is mediated by stabilization of a pathogenic regulator of hepatic lipid accumulation and inflammation. To test this, OTUD1 expression was assessed in human MASLD liver tissues, two independent GEO datasets, and dietary mouse models, and its function was evaluated using systemic *Otud1*-knockout mice and hepatocyte-specific AAV8-TBG-sh*Otud1* silencing in HFMCD-induced steatohepatitis. Using integrated interactome, biochemical, mutagenesis, and transcriptomic approaches, HSP90α was identified as a direct substrate of OTUD1. OTUD1 stabilized HSP90α by removing K48-linked polyubiquitin chains at K283, whereas the catalytically inactive OTUD1-C320S mutant failed to stabilize HSP90α or promote lipid accumulation. Consistent with these findings, *Otud1* deficiency alleviated hepatic steatosis, insulin resistance, and inflammation, and transcriptomic profiling indicated that *OTUD1* activates lipid metabolism and inflammatory pathways. Together, these findings establish the OTUD1–HSP90α axis as a previously unrecognized driver of MASLD progression and support OTUD1 as a potential therapeutic target for metabolic liver disease.

## Results

### OTUD1 expression levels are increased in patients and mice with MASLD

**Fig. 1:**
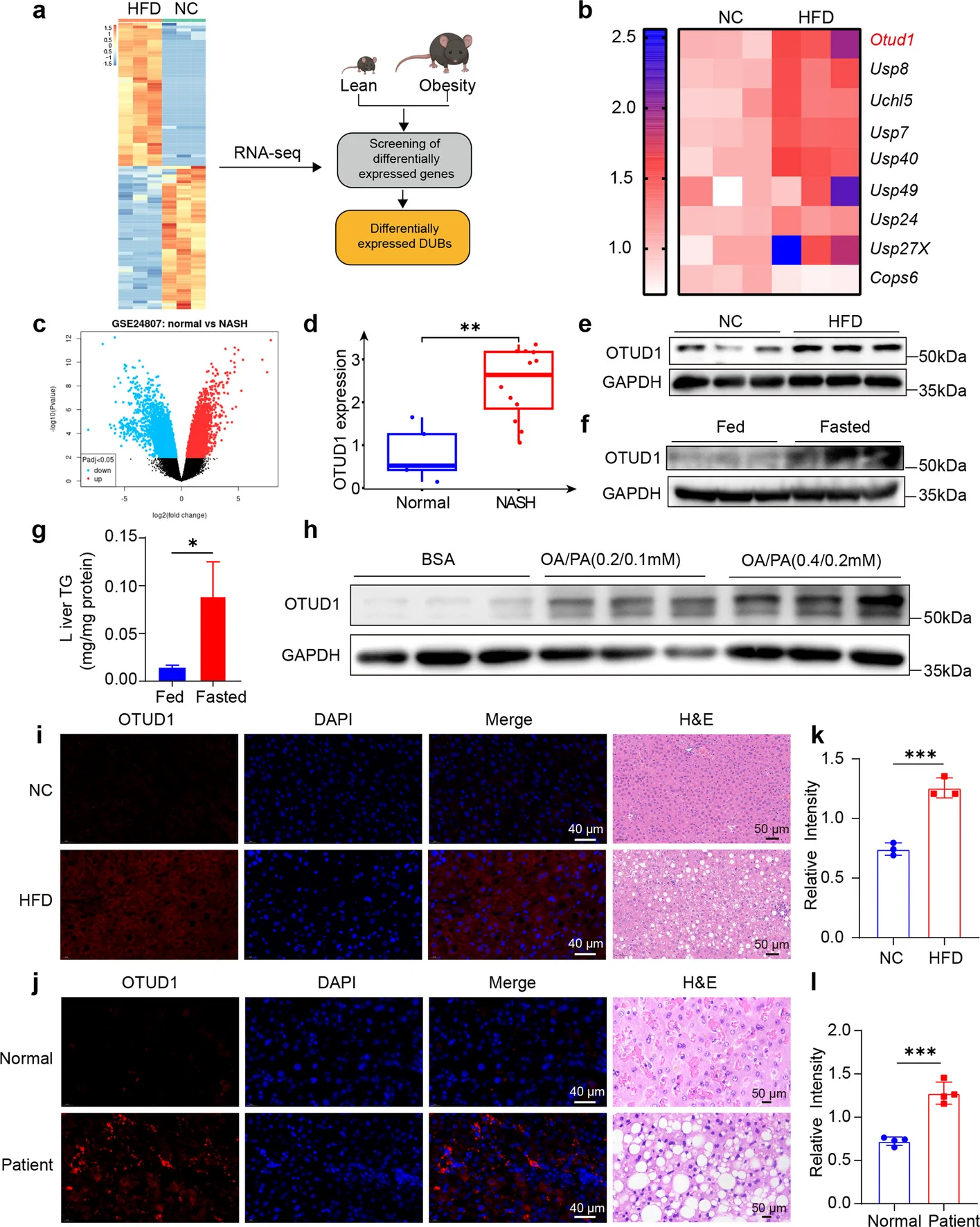
OTUD1 expression levels are increased in patients and mice with MASLD. **a** Heatmap of differentially expressed genes (DEGs) in mouse livers from mice fed normal chow (NC, *n* = 3) or a high-fat diet (HFD, *n* = 3), identifying differentially expressed deubiquitinating enzymes (DUBs). **b** Heatmap of the differentially expressed DUBs (*n* = 3). **c** Volcano plots of liver samples from normal controls (*n* = 5) and patients with metabolic dysfunction-associated steatohepatitis (*n* = 12) from a GEO dataset (GSE24807). **d** Boxplots showing OTUD1 expression levels in the GEO dataset GSE24807. **e** OTUD1 protein levels were measured in livers of mice fed normal chow (NC) or a high-fat diet (HFD) by western blot analysis (*n* = 3). **f** OTUD1 protein levels were measured in livers of fed and fasted mice by western blot analysis (*n* = 3). **g** Liver TG levels in mouse liver under fed and fasted conditions (*n* = 3). **h** Protein levels of OTUD1 in LO2 cells treated with OA/PA at the indicated concentrations. **i** Representative images of immunofluorescence (IF) staining of OTUD1 and H&E staining of liver tissues from mice fed normal chow (NC) or a high-fat diet (HFD) (*n* = 3). Scale bars, 40 µm for IF staining and 50 µm for H&E staining. **j** Representative images of immunofluorescence staining and H&E staining of OTUD1 in liver tissues from patients with MASLD (*n* = 4). Scale bars, 40 µm for immunofluorescence staining and 50 µm for H&E staining. **k**, **l** IF staining quantification from **i** (*n* = 3) and from **j** (*n* = 4). Data are presented as mean ± SD (error bars, SD). *p < 0.05, **p < 0.01, ***p < 0.001; N.S., not significant. Detailed statistical tests are described in the “Statistical analysis” section

High-fat diet (HFD)-fed mice serve as established MASLD models, showing high body weight and blood glucose levels and reduced insulin sensitivity compared with those of controls, along with severe hepatic steatosis [15, 42–44]. To detect the abnormal expression of DUBs in MASLD, unbiased RNA sequencing (RNA-seq) was conducted on liver tissues from HFD- and normal diet-fed mice (Fig. 1a). *Otud1* was among the most upregulated DUBs (Fig. 1b). Furthermore, to explore the potential role of OTUD1 in patients with MASLD, we analyzed a publicly available dataset (GSE24807) comprising 5 normal controls and 12 patients with NASH from the Gene Expression Omnibus (GEO) database. Compared with those in normal controls, hepatic *OTUD1* mRNA levels were significantly higher in patients with NASH (Fig. 1c, d). The same result was observed in another GEO dataset (GSE126848; Fig. S1a, b). Furthermore, hepatic OTUD1 protein levels were consistently upregulated in HFD-fed mice (Fig. 1e). To further assess the tissue specificity of OTUD1 upregulation, immunoblotting analysis was performed on epididymal white adipose tissue (eWAT) and brown adipose tissue (BAT) from HFD-fed mice. Notably, OTUD1 upregulation was not observed in either tissue (Fig. S1c, d), suggesting that HFD-induced OTUD1 elevation may preferentially occur in the liver. In addition, the hepatic OTUD1 protein level was significantly increased under fasting conditions compared with that under fed conditions, with increased liver triglyceride (TG) levels (Fig. 1f, g). Moreover, the OTUD1 protein level was elevated in LO2 cells treated with oleic acid (OA) and palmitic acid (PA) in a dose- (Fig. 1h) and time-dependent manner (Fig. S1e). Immunofluorescence staining demonstrated markedly increased hepatic OTUD1 expression in HFD-fed mice and patients with MASLD compared with their respective controls (Fig. 1i, j). Consistent with these observations, quantitative immunofluorescence analysis further confirmed a significant increase in OTUD1 fluorescence intensity in the livers of HFD-fed mice and patients with MASLD (Fig. 1k, l). Moreover, hepatic OTUD1 expression was positively correlated with NAS scores in both mice and patients, indicating that elevated OTUD1 expression is closely associated with the severity of fatty liver disease (Fig. S1f, g). Collectively, these results suggest that OTUD1 is activated in hepatocytes during MASLD, indicating a potentially crucial role for OTUD1 in MASLD progression.

### Otud1 deletion alleviates HFD-induced hepatic steatosis

**Fig. 2:**
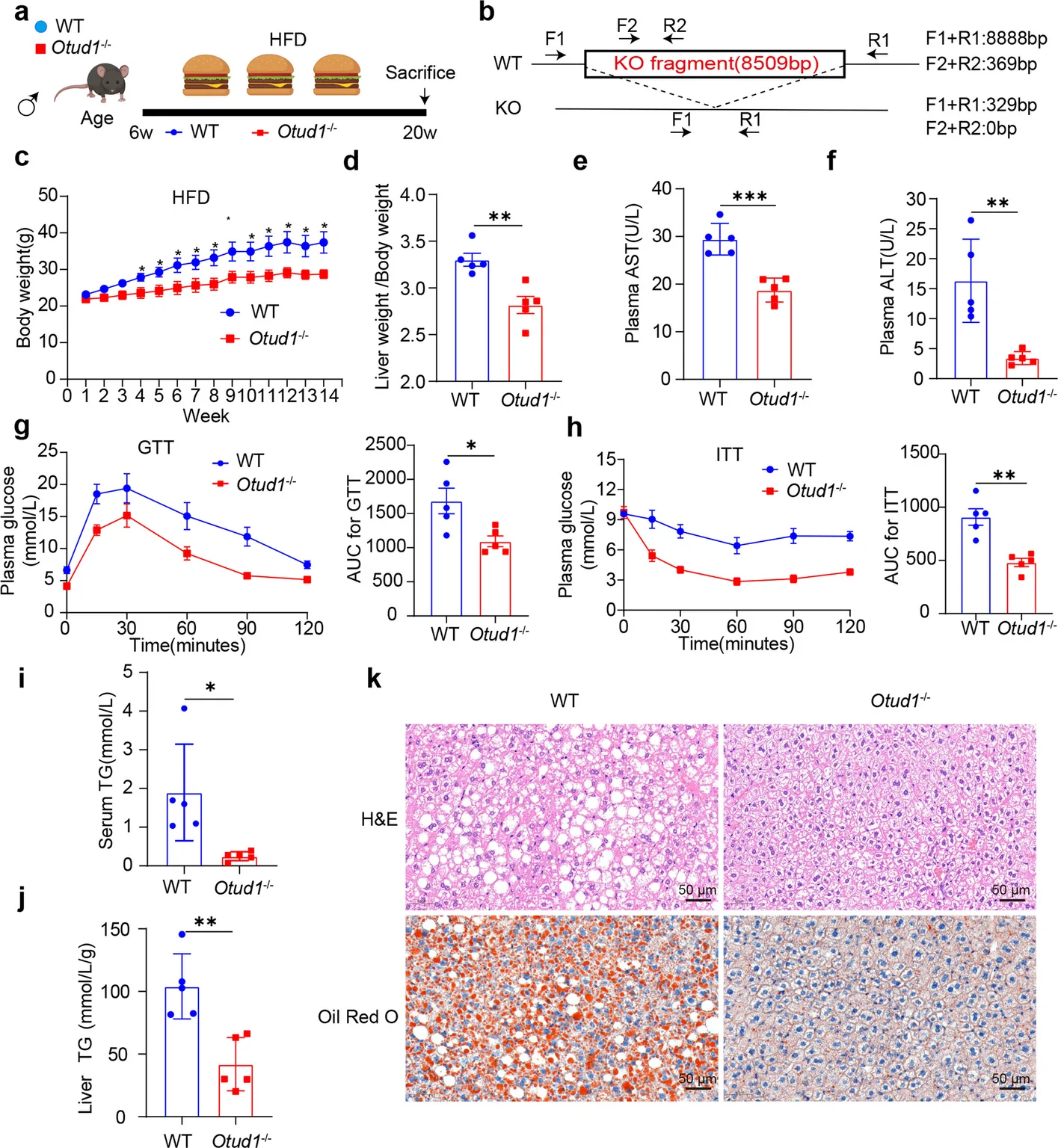
*Otud1* deletion alleviates HFD-induced hepatic steatosis. **a** Schematic representation of WT (n = 5) and *Otud1*^*−/−*^ (n = 5) mice fed an HFD for 14 weeks (Created by BioRender: https://BioRender.com/0ggoq47). **b** Schematic representation of the generation and genotyping of WT and *Otud1*^*−/−*^ mice. **c** Body weights of WT and *Otud1*^*−/−*^ mice fed an HFD for 14 weeks (*n* = 5). **d** Liver weight-to-body weight ratios of WT and *Otud1*^*−/−*^ mice (*n* = 5). **e**, **f** Plasma AST (**e**) and ALT (**f**) levels (*n* = 5). **g** Results of a glucose tolerance test (GTT) in WT and *Otud1*^*−/−*^ mice fed an HFD for 14 weeks and the area under the curve (AUC) for the GTT (n = 5). **h** Insulin tolerance test (ITT) results for WT and *Otud1*^*−/−*^ mice fed an HFD for 14 weeks and the area under the curve (AUC) for the ITT (n = 5). **i** Plasma TG levels (n = 5). **j** Liver TG levels (*n* = 5). **k** H&E and Oil Red O staining of liver sections (*n* = 3). Scale bars, 50 µm for H&E, Oil Red O. Data are presented as mean ± SD (error bars, SD). *p < 0.05, **p < 0.01, ***p < 0.001; N.S., not significant. Detailed statistical tests are described in the “Statistical analysis” section

To further explore the function of *Otud1* in vivo, we utilized CRISPR/Cas9 to construct a targeted deletion in murine *Otud1*. Successful knockout was confirmed by genotyping PCR (Fig. S2a). *Otud1*^*−/−*^ and WT male mice were fed an HFD for 14 weeks (Fig. 2a, b). Genetic ablation of *Otud1* resulted in significant attenuation of HFD-induced metabolic perturbations. Specifically, compared with their WT counterparts, *Otud1*^*−/−*^ mice exhibited reduced body weight gain (Fig. 2c) and a lower liver-to-body weight ratio (Fig. 2d), despite the lack of difference in food intake between the groups (Fig. S2b). This was accompanied by improved liver function, as indicated by significantly lower plasma levels of the hepatic injury markers AST and ALT (Fig. 2e, f). Furthermore, *Otud1* deficiency enhanced systemic glucose homeostasis, as demonstrated by improvements in glucose tolerance (GTT) and insulin sensitivity (ITT) (Fig. 2g, h). Crucially, *Otud1* deletion markedly improved lipid homeostasis, as evidenced by reduced plasma triglyceride (TG) and total cholesterol (TC) levels (Fig. 2i and Fig. S2c), as well as decreased hepatic TG levels (Fig. 2j). Consistently, histological analyses revealed markedly attenuated hepatic steatosis, as demonstrated by H&E and Oil Red O staining (Fig. 2k). Correspondingly, quantitative analyses showed significantly lower histological scores (Fig. S2d) and Oil Red O-positive areas (Fig. S2e) in *Otud1*-deficient mice. In line with these metabolic improvements, compared with WT mice, HFD-fed *Otud1*^*−/−*^ mice exhibited smaller adipocytes in BAT, eWAT, and iWAT (Fig. S2f). Collectively, these data demonstrate that *Otud1* deletion effectively alleviates HFD-induced hepatic steatosis and metabolic dysfunction.

### Otud1 deletion alleviates HFMCD-induced hepatic steatohepatitis

**Fig. 3:**
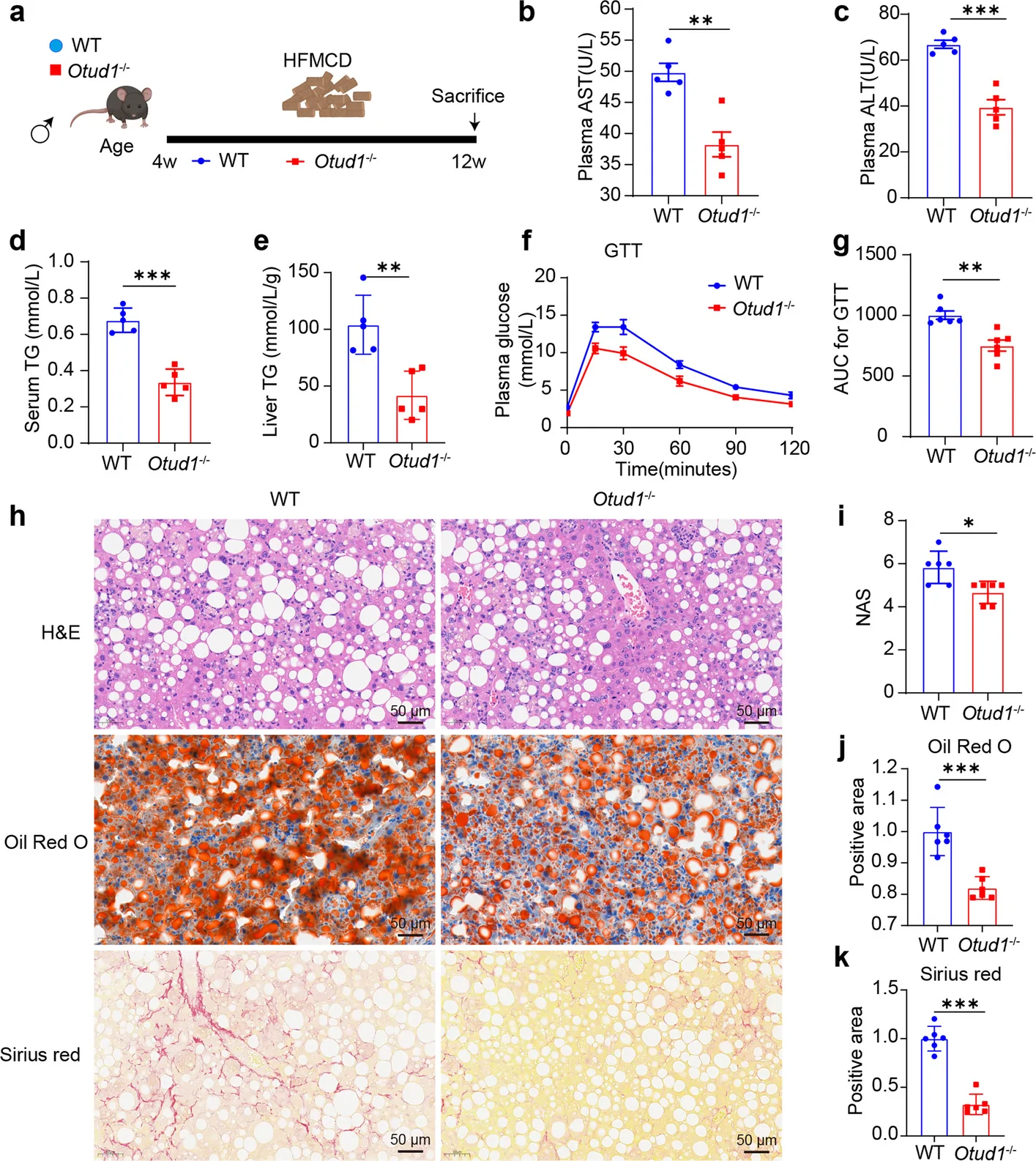
*Otud1* deletion alleviates HFMCD-induced hepatic steatohepatitis. **a** Schematic representation of WT (n = 6) and *Otud1*^*−/−*^ (*n* = 6) mice fed an HFMCD diet for 8 weeks (Created by BioRender: https://BioRender.com/0ggoq47). **b**, **c** Plasma AST (**b**) and ALT (**c**) levels (*n* = 5). **d** Plasma triglyceride (TG) levels (*n* = 5). **e** Liver triglyceride (TG) levels (*n* = 5). **f** Glucose tolerance test (GTT) results for WT and *Otud1*^*−/−*^ mice fed an HFMCD diet for 8 weeks (*n* = 6). **g** Area under the curve (AUC) for the GTT. **h** H&E, Oil Red O staining and Sirius red staining of liver sections, followed by quantitative analysis using ImageJ for H&E, Oil Red O staining and Sirius red (*n* = 6). Scale bars, 50 µm for H&E, Oil Red O staining and Sirius red staining. **i**–**k** NAS scores in livers from WT and *Otud1*^*−/−*^ mice fed an HFMCD and quantitative analysis of Oil Red O staining and Sirius red staining (*n* = 6). Data are presented as mean ± SD (error bars, SD). *p < 0.05, **p < 0.01, ***p < 0.001; N.S., not significant. Detailed statistical tests are described in the “Statistical analysis” section

To investigate the role of *Otud1* in diet-induced steatohepatitis, we fed 4-week-old male WT and *Otud1*^*−/−*^ mice a high-fat, methionine-restricted, choline-deficient diet (HFMCD) for 8 weeks (Fig. 3a). Successful *Otud1* deletion was confirmed in metabolic tissues, including the liver, eWAT, and inguinal iWAT (Fig. S3a–c). *Otud1* deficiency markedly attenuated the development of steatohepatitis. Although food intake and body weight were comparable between WT and *Otud1*^−/−^ mice throughout the dietary challenge (Fig. S3d, e), *Otud1* deletion alleviated liver injury, as indicated by decreased plasma AST and ALT levels (Fig. 3b, c). Furthermore, *Otud1*^−/−^ mice exhibited reduced plasma triglyceride (TG) and total cholesterol (TC) levels (Fig. 3d and Fig. S3f), as well as decreased hepatic TG levels (Fig. 3e), compared with WT mice. *Otud1*^*−/−*^ mice showed significant improvements in glucose tolerance (Fig. 3f, g). Histological analyses further revealed significant reductions in hepatic lipid accumulation and fibrosis in *Otud1*^*−/−*^ mice, as demonstrated by Oil Red O and Sirius red staining, respectively (Fig. 3h). Consistent with these histological findings, quantitative analyses showed significantly lower NAS values (Fig. 3i), reduced Oil Red O-positive areas (Fig. 3j), and decreased Sirius red-positive areas (Fig. 3k) in *Otud1*^*−/−*^ mice compared with WT mice. Collectively, these data suggest that *Otud1* deletion protects against HFMCD-induced hepatic steatohepatitis.

### OTUD1 interacts and colocalizes with HSP90α in hepatocytes

**Fig. 4:**
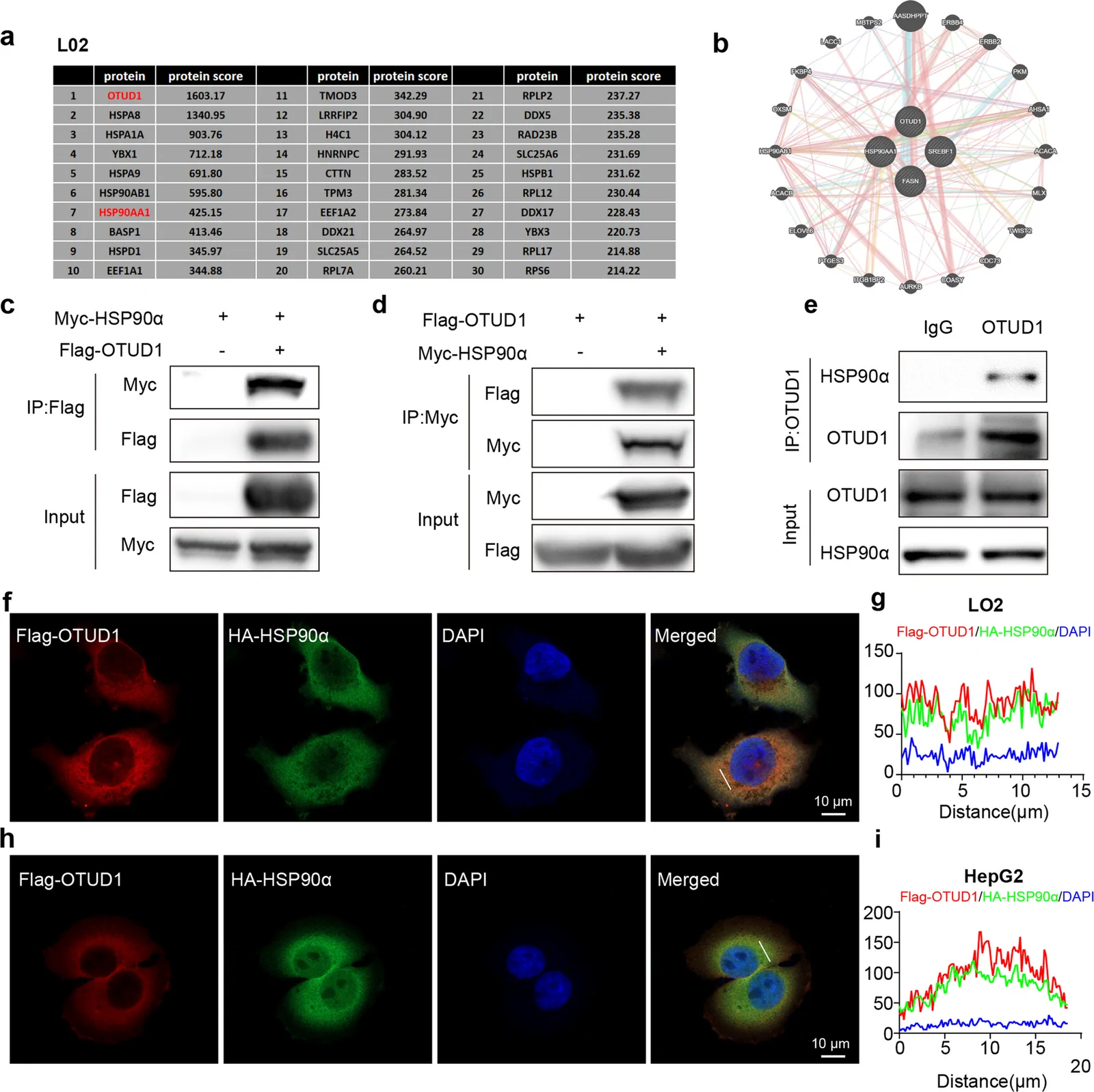
OTUD1 interacts and colocalizes with HSP90α. **a** Representative mass spectrometry analysis of proteins coimmunoprecipitated with OTUD1 in LO2 cells. **b** Protein–protein interaction (PPI) network analysis based on GeneMANIA and genes involved in the indicated pathways. **c**, **d** Exogenous co-IP assays were performed to assess the interaction between OTUD1 and HSP90α in HEK293T cells transfected with Flag-tagged *OTUD1* and Myc-tagged *HSP90α*. **e** Endogenous co-IP assays were performed to assess the interaction between OTUD1 and HSP90α in Huh7 cells. (f–i) OTUD1 colocalizes with HSP90α. **f** LO2 cells were transiently transfected with Flag-tagged OTUD1 and HA-tagged HSP90α and subjected to immunofluorescence staining. **g** The fluorescence intensity profiles of Flag-OTUD1 (red), HA-HSP90α (green), and DAPI (blue) along the indicated line in LO2 cells. **h** HepG2 cells were transiently transfected with Flag-tagged OTUD1 and HA-tagged HSP90α and subjected to immunofluorescence staining. **i** The fluorescence intensity profiles of Flag-OTUD1 (red), HA-HSP90α (green), and DAPI (blue) along the indicated line in HepG2 cells

To elucidate the molecular mechanism through which OTUD1 promotes MASLD, we sought to identify its interacting partners. Coimmunoprecipitation coupled with mass spectrometry (Co-IP/MS) in LO2 cells (Fig. 4a) and HEK293T cells (Fig. S4a) revealed that HSP90α is strongly associated with OTUD1. Protein–protein interaction network analysis further supported the connectivity between OTUD1 and pathways involving HSP90α (Fig. 4b). To further validate the interaction between OTUD1 and HSP90α, coimmunoprecipitation assays were performed at both exogenous and endogenous levels. Exogenous Co-IP analysis revealed that overexpressed OTUD1 physically associated with HSP90α (Fig. 4c, d), whereas endogenous Co-IP further confirmed the interaction between OTUD1 and HSP90α under physiological expression conditions (Fig. 4e), excluding the possibility that this association was merely an artifact of ectopic overexpression. To further determine their intracellular spatial relationship, immunofluorescence colocalization analysis was performed in both LO2 and HepG2 cells. The results demonstrated substantial colocalization of OTUD1 and HSP90α within cells (Fig. 4f–i), providing additional evidence for their physical association in hepatocytes. Collectively, these findings confirm that HSP90α is an OTUD1-interacting protein at both the protein interaction and subcellular localization levels.

### OTUD1 deubiquitinates and stabilizes HSP90α by removing its K48-linked ubiquitin chains

**Fig. 5:**
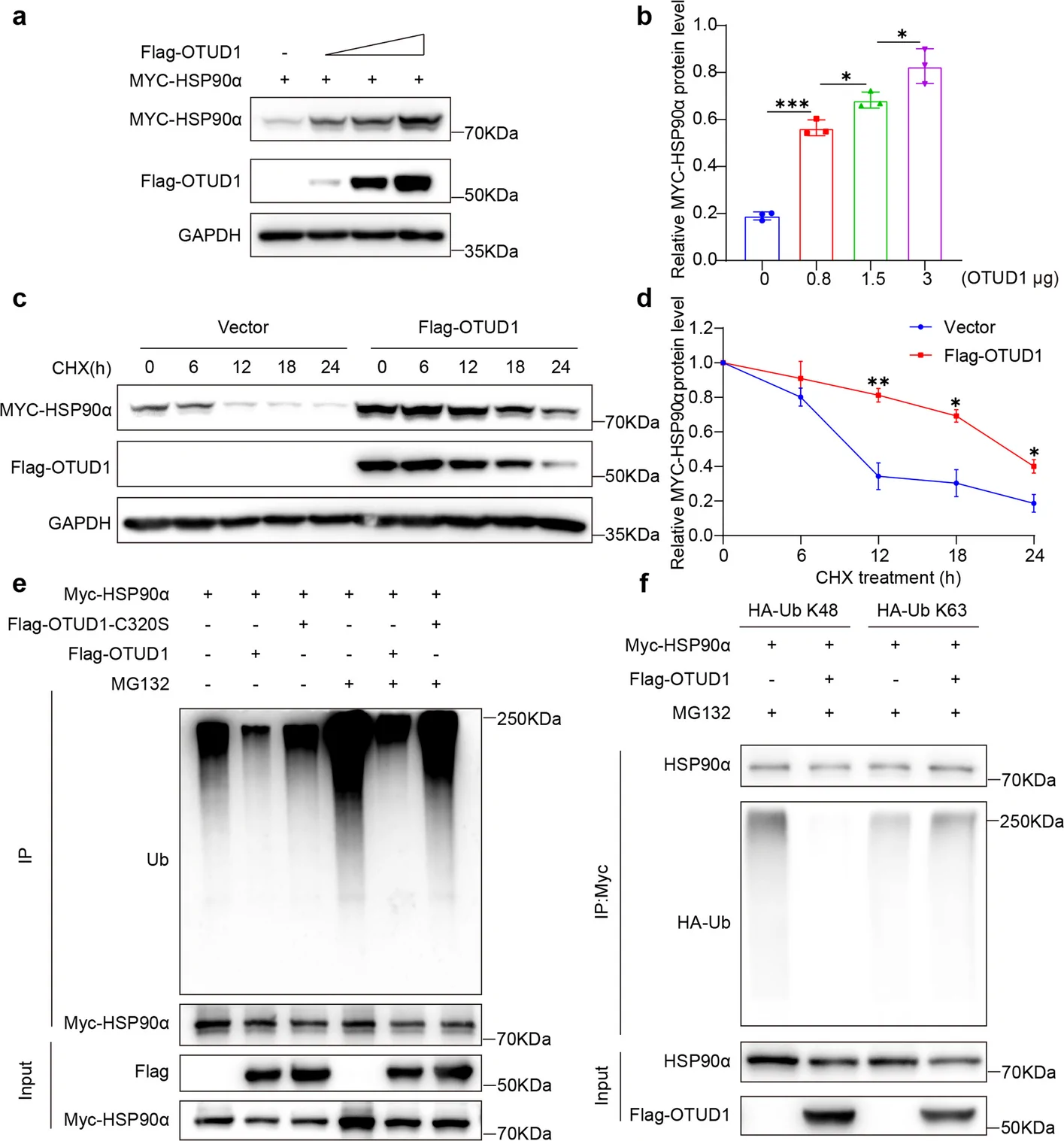
OTUD1 deubiquitinates and stabilizes HSP90α by removing its K48-linked ubiquitin chains. **a** OTUD1 overexpression increased HSP90α protein levels in a dose-dependent manner. Western blot analysis of HSP90α abundance in HEK293T cells transfected with increasing amounts of Flag-tagged OTUD1. **b** Quantification of Myc-HSP90α from the experiments in (**a**). **c** Half-life analysis of the abundance of HSP90α in HEK293T cells over the indicated time periods in the presence of CHX (25 µg/mL). **d** Quantification of Myc-HSP90α from the experiments in (**c**). **e** OTUD1 overexpression decreases HSP90α ubiquitination. HEK293T cells were transfected with plasmids encoding HSP90α, OTUD1, or the catalytically inactive mutant OTUD1-C320S, as indicated. The cells were then treated with MG132 (10 µM) for 12 h, followed by immunoprecipitation (IP) and immunoblotting (IB) using the indicated antibodies. **f** Identification of the type of HSP90α deubiquitination by OTUD1. Data are presented as mean ± SD (error bars, SD). *p < 0.05, **p < 0.01, ***p < 0.001; N.S., not significant. Detailed statistical tests are described in the “Statistical analysis” section

Having established the physical interaction between OTUD1 and HSP90α, we next investigated whether OTUD1 regulates HSP90α protein stability through a ubiquitin-dependent mechanism. In HEK293T cells, dose-dependent cotransfection assays revealed that increasing OTUD1 expression progressively increased the abundance of the HSP90α protein (Fig. 5a, b), indicating that OTUD1 positively regulates the protein level of HSP90α. Consistent with these findings, the results of the cycloheximide chase assays demonstrated that OTUD1 overexpression markedly prolonged the half-life of HSP90α (Fig. 5c, d), suggesting that OTUD1 plays a role in restraining HSP90α degradation.

Given that OTUD1 is a deubiquitinating enzyme, we examined whether OTUD1 stabilizes HSP90α by modulating its ubiquitination status. Ubiquitination assays revealed that OTUD1 overexpression substantially reduced the overall ubiquitination of HSP90α (Fig. 5e). Importantly, compared with wild-type OTUD1, the catalytically inactive mutant OTUD1-C320S markedly impaired the ability to remove ubiquitin from HSP90α (Fig. 5e). These results indicate that OTUD1-mediated stabilization of HSP90α largely depends on its deubiquitinase activity rather than merely on substrate binding or a scaffolding effect.

To define the ubiquitin-chain specificity regulated by OTUD1, we assessed the effects of distinct ubiquitin linkages on HSP90α. OTUD1 preferentially removed K48-linked polyubiquitin chains from HSP90α, with relatively limited effects on K63-linked ubiquitination (Fig. 5f). Because K48-linked polyubiquitination is a canonical signal for proteasome-dependent protein degradation, these data support the conclusion that OTUD1 protects HSP90α from ubiquitin–proteasome pathway-mediated turnover. Finally, site-directed mutagenesis of candidate lysine residues in HSP90α followed by ubiquitination immunoprecipitation assays revealed that mutation of lysine 283 markedly impaired OTUD1-mediated deubiquitination of HSP90α, identifying K283 as a critical ubiquitination site regulated by OTUD1 (Fig. S5a, b).

Previous studies have established STUB1 as an E3 ubiquitin ligase for HSP90α, which we confirmed in our experimental system (Fig. S5c). STUB1 overexpression significantly increased HSP90α ubiquitination, whereas the coexpression of OTUD1 markedly attenuated STUB1-induced HSP90α ubiquitination. These findings suggest that STUB1 promotes the ubiquitin modification and destabilization of HSP90α, whereas OTUD1 counteracts this process by deubiquitinating HSP90α.

Taken together, these results demonstrate that OTUD1 acts as a deubiquitinase for HSP90α by removing K48-linked polyubiquitin chains associated with HSP90α K283. This prevents proteasome-dependent degradation, enhances the stability of the HSP90α protein, and provides a mechanistic basis through which OTUD1 increases the abundance of HSP90α to facilitate inflammatory signaling activation and hepatic lipid accumulation.

### OTUD1 promotes lipid accumulation through HSP90α in hepatocytes

Based on our findings in clinical samples and animal models, we demonstrated that OTUD1 plays a pro-pathogenic role in MASLD/MASH progression and identified HSP90α as a major OTUD1-interacting protein and downstream effector. However, whether the OTUD1–HSP90α axis directly regulates hepatocyte lipid metabolism remains unclear. To address this question, we generated stable *OTUD1*-overexpressing and *OTUD1*-knockout LO2 cell lines using lentiviral overexpression and sgRNA-mediated knockout strategies, respectively (Fig. S6a, b).

We first established an in vitro hepatocyte steatosis model. OA/PA treatment was evaluated across multiple hepatic cell lines, and cell viability assays defined nontoxic concentrations suitable for subsequent experiments in HepG2, LO2, and Huh7 cells (Fig. S6c–f). Under these conditions, OA/PA robustly induced lipid accumulation, as confirmed by Oil Red O staining and quantitative analysis in HepG2 and LO2 cells (Fig. S6g–j). Based on its low cytotoxicity and strong steatogenic effect, OA/PA treatment at 400/200 µM was selected for subsequent experiments.

**Fig. 6:**
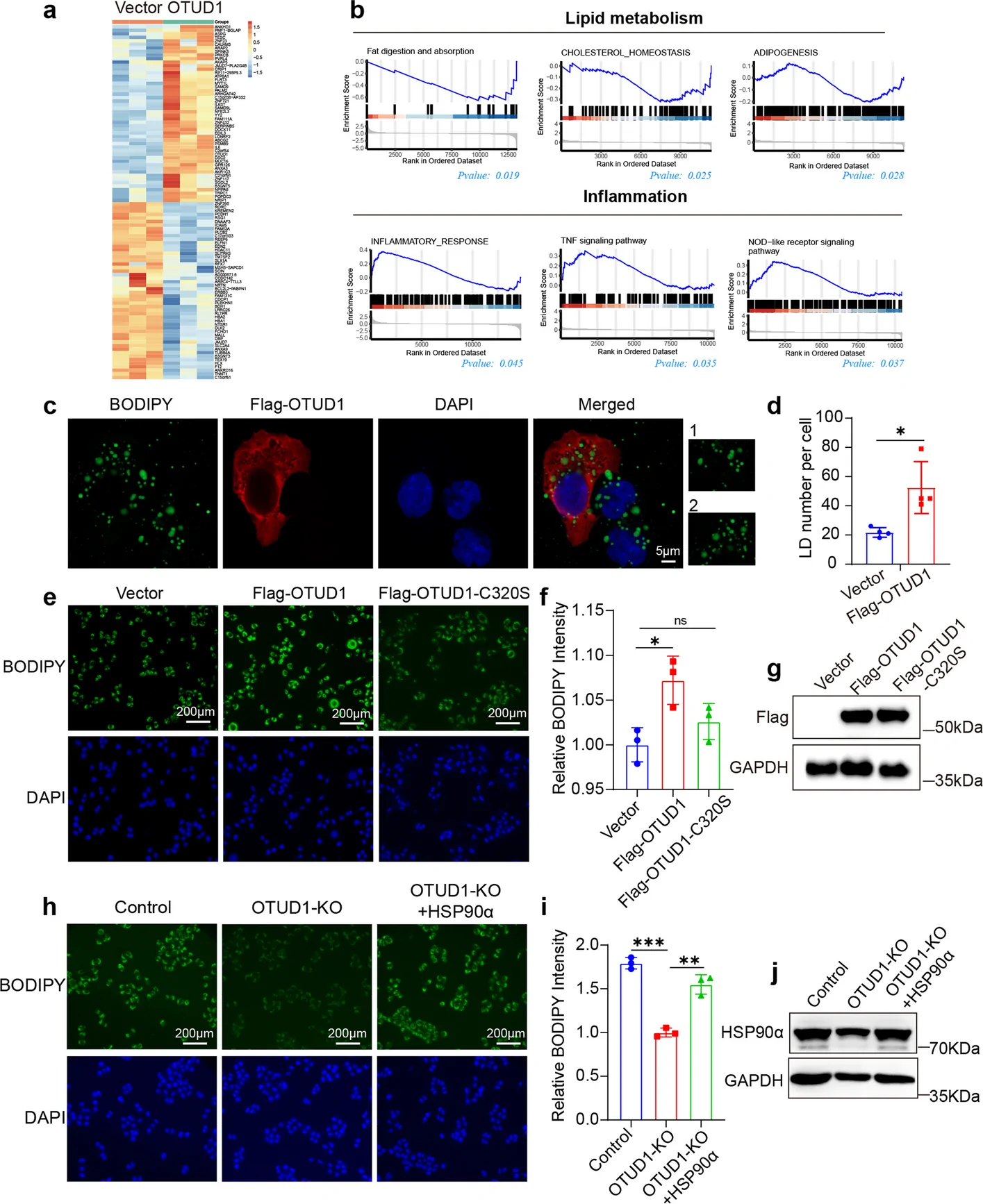
OTUD1 promotes lipid accumulation through HSP90α in hepatocytes. **a** Heatmap of the differentially expressed genes (DEGs) in OA/PA-treated LO2 cells transfected with vector or Flag-OTUD1. **b** Gene set enrichment analysis showing the activation of pathways associated with lipid metabolism and inflammation in the OTUD1-overexpressing group compared with the control group. **c** Confocal images of Flag-OTUD1-transfected LO2 cells after OA/PA treatment; the lipid droplets (LDs) were stained with BODIPY 493/503. Inset 1 in (**c**) depicts an untransfected cell, and inset 2 depicts a cell expressing Flag-OTUD1 (*n* = 4). Scale bars, 5 µm for confocal microscopy images. **d** Quantitative data derived from similar images as in (**c**) across three independent experiments, including the number of LDs per cell (*n* = 4). **e** BODIPY staining of OA/PA-treated stable LO2 cell lines expressing vector (*n* = 3), Flag-OTUD1 (*n* = 3), or Flag-OTUD1-C320S (*n* = 3). The nuclei were counterstained with DAPI. Scale bars, 200 µm. **f** Quantitative BODIPY staining derived from similar images as in (**e**) via a high-content imaging system (*n* = 3). **g** OTUD1 protein levels in LO2 cells transfected with vector, Flag-OTUD1-C320S or Flag-OTUD1. **h** BODIPY staining in OA/PA-treated LO2 cells (WT), OTUD1-knockout LO2 cells (OTUD1-KO) and HSP90α-reconstituted OTUD1-knockout cells (*n* = 3). Scale bars, 200 µm for BODIPY staining images. **i** Quantitative BODIPY staining derived from similar images as in (**h**) via a high-content imaging system (*n* = 3). **j** The protein levels of HSP90α in WT and OTUD1-knockout LO2 cells and HSP90α-reconstituted in OTUD1-knockout cells. Data are presented as mean ± SD (error bars, SD). *p < 0.05, **p < 0.01, ***p < 0.001; N.S., not significant. Detailed statistical tests are described in the “Statistical analysis” section

To investigate the downstream consequences of OTUD1 activation in hepatocytes, we performed transcriptomic profiling in OA/PA-treated LO2 cells overexpressing OTUD1. OTUD1 markedly altered the cellular transcriptional program, with GSEA revealing significant enrichment of pathways involved in lipid metabolism and inflammatory responses (Fig. 6a, b). In line with these findings, transient overexpression of OTUD1 increased lipid droplet accumulation, as shown by enhanced BODIPY staining after OA/PA exposure (Fig. 6c, d); a similar effect was also observed in the absence of OA/PA treatment (Fig. S6k). We next confirmed these observations in stable cell models. Stable overexpression of OTUD1 aggravated OA/PA-induced lipid accumulation (Fig. 6e–g). Importantly, compared with wild-type OTUD1, the catalytically inactive OTUD1-C320S mutant was markedly less effective at promoting lipid accumulation (Fig. 6e–g), indicating that the pro-steatotic effect of OTUD1 is dependent on its deubiquitinase activity. Conversely, OTUD1 knockout significantly reduced OA/PA-induced lipid accumulation (Fig. 6h–j). Notably, restoration of HSP90α expression in OTUD1-KO cells rescued lipid accumulation to levels comparable to those in wild-type cells (Fig. 6h–j), further supporting HSP90α as a key downstream effector of OTUD1 in hepatocellular lipid accumulation.

Collectively, our results not only corroborate the known functions of HSP90α in lipogenesis but also provide a novel mechanistic layer through the identification of OTUD1 as a crucial upstream deubiquitinating stabilizer. This is consistent with previous reports demonstrating the pro-lipogenic function of HSP90 [39, 40].

### Otud1 deletion alleviates lipogenesis and inflammation in mice

**Fig. 7:**
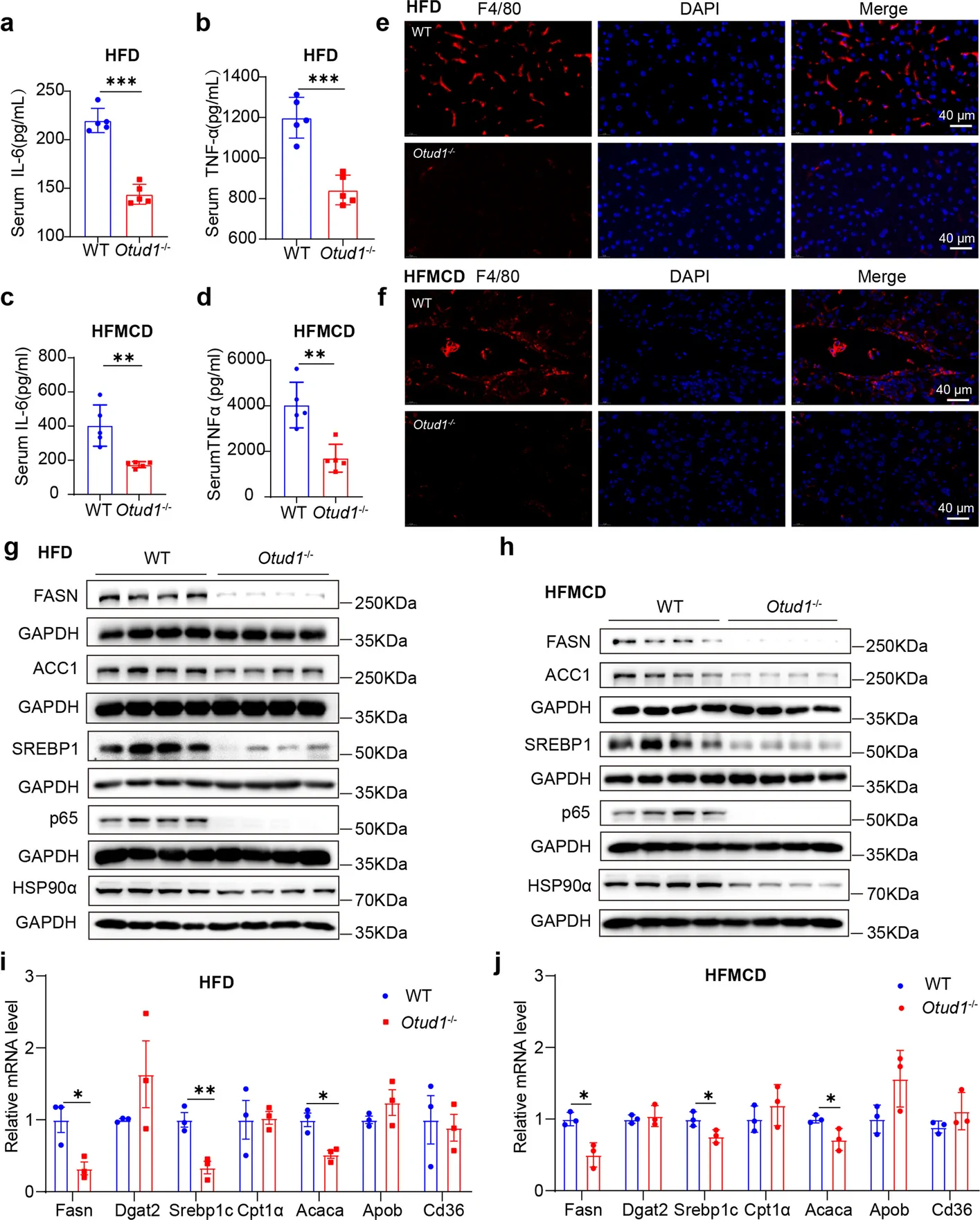
*Otud1* deletion alleviates lipogenesis and inflammation in mice. **a**, **b** Serum IL-6 and TNF-α levels in WT and *Otud1*^*−/−*^ mice fed an HFD (*n* = 5). **c**, **d** Serum IL-6 and TNF-α levels in WT and *Otud1*^*−/−*^ mice fed an HFMCD diet (*n* = 5). **e**, **f** Immunofluorescence detection of F4/80 expression in the livers of WT and *Otud1*^*−/−*^ mice fed an HFD (**e**) or an HFMCD diet (**f**) (*n* = 3). Scale bars, 40 µm for immunofluorescence (IF) staining. (**g**, **h**) Representative immunoblotting analysis of lipid metabolism-related proteins (FASN, ACC1, and SREBP1) and other proteins, such as p65 and HSP90α, in liver tissues from WT and *Otud1*^*−/−*^ mice fed an HFD or an HFMCD diet (*n* = 4). **i**, **j** Gene expression analysis of lipid metabolism-related genes in the livers of WT and *Otud1*^*−/−*^ mice fed an HFD or an HFMCD diet (*n* = 3). Data are presented as mean ± SD (error bars, SD). *p < 0.05, **p < 0.01, ***p < 0.001; N.S., not significant. Detailed statistical tests are described in the “Statistical analysis” section

Given the in vitro findings presented above, the transcriptomic enrichment of inflammatory pathways, and the previously established pro-inflammatory role of HSP90α [14, 39], we investigated the in vivo effects of *Otud1* deficiency on systemic inflammation and hepatic lipogenesis. Following HFD or HFMCD challenge, *Otud1*^*−/−*^ mice exhibited markedly reduced systemic inflammation, as evidenced by lower serum levels of IL-6 and TNF-α (Fig. 7a–d) and significantly attenuated F4/80-positive macrophage infiltration in the liver (Fig. 7e, f). Further phenotypic characterization revealed a shift in hepatic macrophage polarization toward an anti-inflammatory state, marked by upregulation of the M2 marker CD206 and downregulation of the M1 marker CD86 (Fig. S7a–d). Importantly, this anti-inflammatory reprogramming was cell-intrinsic, as LPS-stimulated bone marrow-derived macrophages (BMDMs) from *Otud1*^*−/−*^ mice similarly displayed diminished CD86 expression and downregulated Il1b mRNA levels (Fig. S7e, f).

Mechanistically, in line with the finding that HSP90α acts as a functional target of OTUD1, hepatic HSP90α protein levels were markedly reduced in *Otud1*^*−/−*^ mice. This was accompanied by a concurrent decrease in p65 abundance, confirming that *Otud1* deficiency suppressed HSP90α-associated inflammatory signaling in vivo (Fig. 7g, h). In parallel with the attenuation of inflammation, *Otud1* deficiency markedly reduced the protein levels of the key lipogenic factors FASN, ACC1, and SREBP1 in HFD-fed mice (Fig. 7g), with similar changes observed under HFMCD conditions (Fig. 7h). Consistently, the hepatic mRNA levels of *Fasn*, *Srebp1c*, and *Acaca* were significantly decreased in *Otud1*^*−/−*^ mice under both HFD and HFMCD conditions (Fig. 7i, j).

Collectively, these in vivo results demonstrate that *Otud1* deletion effectively ameliorates hepatic steatosis through a dual mechanism: the suppression of macrophage-mediated inflammatory responses and the deactivation of hepatic lipogenic transcriptional programs.

### Hepatic Otud1 knockdown by AAV8-TBG-shOtud1 alleviates diet-induced steatohepatitis in mice

**Fig. 8:**
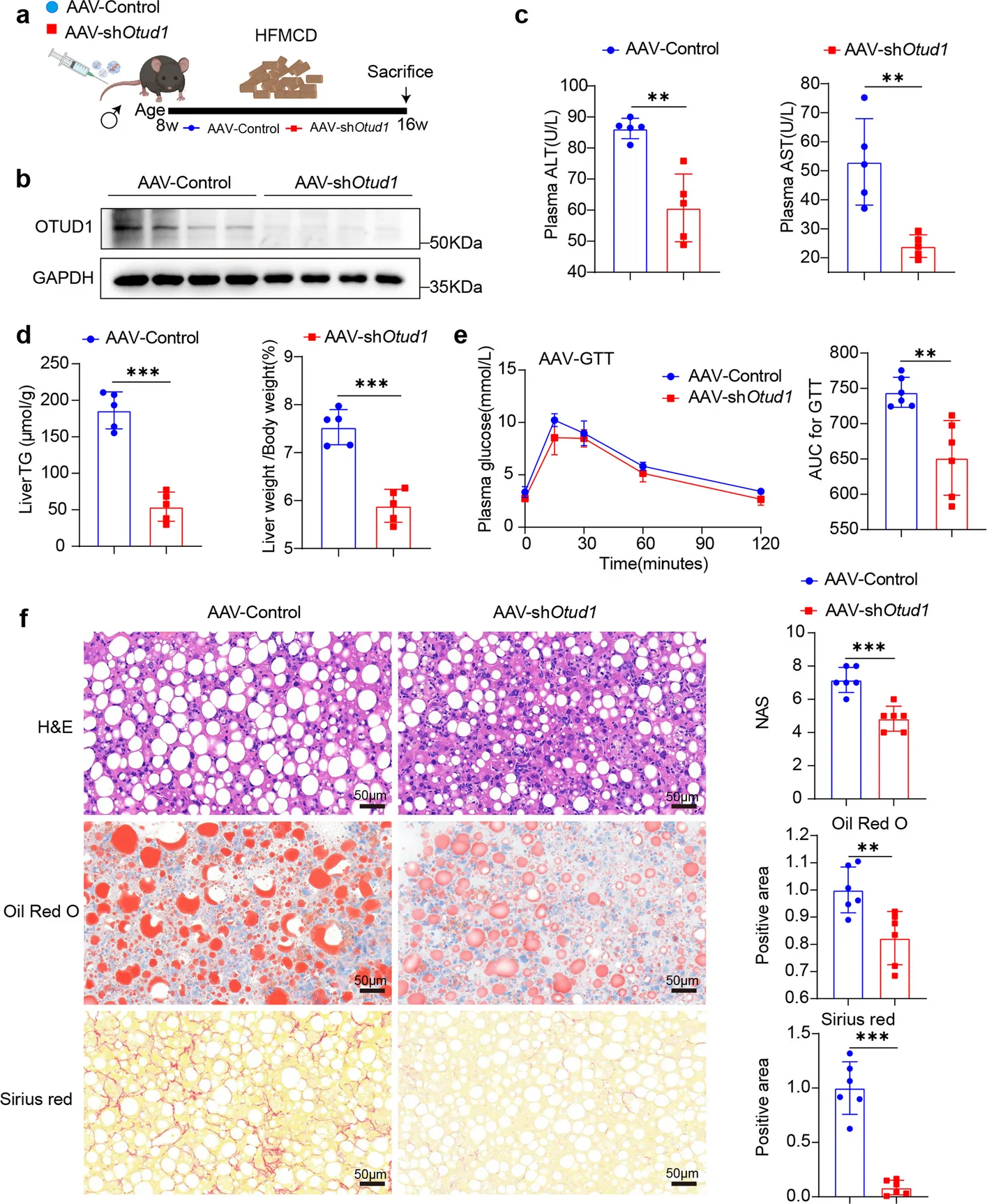
Hepatic *Otud1* knockdown by AAV8-TBG-sh*Otud1* alleviates diet-induced steatohepatitis in mice. **a** Schematic representation of AAV-Control (*n* = 6) and AAV-sh*Otud1* (*n* = 6) mice fed an HFMCD diet for 8 weeks (Created by BioRender: https://BioRender.com/0ggoq47). **b** The protein levels of OTUD1 in the livers of AAV-Control (*n* = 4) and AAV-sh*Otud1* (*n* = 4) mice. **c** Plasma AST and ALT levels (*n* = 5). **d** Liver triglyceride (TG) levels and the liver-to-body weight ratio (*n* = 5). **e** Glucose tolerance test (GTT) results for AAV-Control (*n* = 6) and AAV-sh*Otud1* (*n* = 6) mice fed an HFMCD diet for 8 weeks and the area under the curve (AUC) for the GTT (*n* = 6). **f** H&E staining, Oil Red O staining and Sirius red staining of liver sections, followed by quantitative analysis using ImageJ with Oil Red O staining, Sirius red staining, and NAS scoring in livers from AAV-Control and AAV-sh*Otud1* mice fed an HFMCD diet (*n* = 6). Scale bars, 50 µm for H&E, Oil Red O and Sirius red. Data are presented as mean ± SD (error bars, SD). *p < 0.05, **p < 0.01, ***p < 0.001; N.S., not significant. Detailed statistical tests are described in the “Statistical analysis” section

Given the protection conferred by global *Otud1* deletion, we evaluated whether liver-specific *Otud1* knockdown could serve as a therapeutic strategy for MASH using AAV8-TBG-sh*Otud1* in HFMCD-fed wild-type mice (Fig. 8a, b). In the absence of altered food intake or body weight (Fig. S8a, b), AAV8-mediated *Otud1* silencing significantly attenuated HFMCD-induced liver injury and metabolic dysfunction, as shown by reduced liver-to-body weight ratios, decreased hepatic triglyceride content, improved glucose tolerance, and decreased plasma ALT/AST levels (Fig. 8c–e). Histological evaluation further confirmed the marked alleviation of both hepatic steatosis and fibrosis progression (Fig. 8f).

Mechanistically, liver-specific *Otud1* knockdown effectively suppressed both lipogenic and inflammatory cascades. *Otud1* silencing dampened hepatic inflammation, as evidenced by reduced serum IL-6 and TNF-α levels and decreased intrahepatic F4/80-positive macrophage infiltration (Fig. S8c, d). Additionally, the protein levels of the key lipogenic regulators—FASN, ACC1, and SREBP1—were substantially downregulated (Fig. S8e). Crucially, hepatic HSP90α and p65 protein levels decreased concurrently (Fig. S8e), further supporting the notion that therapeutic targeting of *Otud1* mitigates MASH pathology, at least in part, through modulation of the HSP90α–p65 signaling axis.

Collectively, these data provide compelling proof-of-concept evidence supporting liver-directed *Otud1* inhibition as a viable therapeutic approach for MASLD/MASH. Mechanistically, AAV8-mediated *Otud1* silencing impedes steatohepatitis progression through the coordinated suppression of HSP90α–p65-dependent inflammatory signaling and hepatic lipogenic activation, highlighting the OTUD1–HSP90α axis as a plausible and druggable therapeutic target.

## Discussion

In this study, we identified OTUD1 as a previously unrecognized pathogenic deubiquitinase in MASLD/MASH that promotes disease progression by stabilizing HSP90α. Integrating evidence from human liver specimens, independent transcriptomic cohorts, diet-induced mouse models, hepatocyte steatosis assays, and liver-directed knockdown experiments, we established an OTUD1–HSP90α regulatory axis that links deubiquitinase-mediated proteostasis to hepatic lipid dysregulation and inflammatory activation. Mechanistically, we defined HSP90α as a direct substrate and functional downstream effector of OTUD1. OTUD1 interacts with HSP90α and stabilizes it by removing K48-linked polyubiquitin chains at lysine 283, thereby preventing its proteasome-dependent degradation. Together, these findings indicate that OTUD1 drives MASLD/MASH progression, at least in part, by sustaining HSP90α-dependent inflammatory and lipogenic programs.

Our findings substantially enrich the functional paradigm of DUBs in metabolic liver diseases. Whereas DUBs such as USP10 and USP29 counteract steatohepatitis, OTUD1 operates alongside USP14, USP15, RPN11, USP24, and USP7 as a potent driver of disease progression [14, 15, 21–23, 45–47]. Our findings place OTUD1 within this pro-pathogenic DUB category in MASLD/MASH. Notably, both OTUD1 and USP14 have been linked to HSP90α stabilization, suggesting that HSP90α may serve as a convergent proteostatic hub regulated by distinct DUBs under metabolic stress. This convergence highlights the importance of substrate-selective deubiquitination in shaping hepatic stress responses and supports the broader DUB–substrate network as a potentially druggable layer in MASLD/MASH pathogenesis.

However, the biological function of a given DUB is not necessarily uniform across liver diseases but may instead depend on the pathological context, dominant cellular stress, and available substrate repertoire. This concept is particularly relevant to OTUD1 because a previous study reported that OTUD1 protects against hepatic ischemia/reperfusion injury by stabilizing NRF2 [48]. We propose that OTUD1 functions in a context- and substrate-dependent manner. In acute oxidative injury, where the dominant stress is ROS, OTUD1-mediated NRF2 stabilization is protective. In chronic metabolic inflammation, OTUD1 predominantly stabilizes HSP90α to drive lipogenesis and inflammation. Consistent with this interpretation, hepatic NRF2 protein levels did not decrease in Otud1^−/−^ mice in our HFD/HFMCD models (data not shown), suggesting that suppression of NRF2 is unlikely to be the primary mechanism underlying the phenotype observed in our MASLD models. This dual-substrate model reconciles the divergent literature and suggests that OTUD1 inhibitor design in MASLD should prioritize selectivity for the HSP90α-stabilizing function without disrupting the beneficial NRF2-stabilizing function.

The limitations of this study include the following. First, all in vivo experiments were performed in male mice; given the well-documented sexual dimorphism in MASLD [49–51], future studies in female mice are warranted. Second, energy expenditure, physical activity, and thermogenesis were not directly assessed using metabolic cage analysis; therefore, the precise mechanism by which *Otud1* deficiency reduces body weight gain remains to be elucidated. Third, although our qRT-PCR results suggest that OTUD1 modulates hepatic inflammatory signaling in macrophages, the precise tissue-specific contribution to the observed systemic cytokine reduction was not directly dissected. Addressing these limitations will be an important focus of our future work.

In summary, our study identifies the deubiquitinase OTUD1 as a previously unrecognized promoter of MASLD/MASH that stabilizes HSP90α through the removal of K48-linked polyubiquitin chains at K283, antagonizing the E3 ligase STUB1. The OTUD1–HSP90α regulatory axis delineated here represents a novel and potentially druggable pathway for the treatment of metabolic liver disease.

## Materials and methods

### Antibodies and plasmids

GAPDH (1:5000, cat# AC002, ABclonal), OTUD1 (1:1000, cat# 29,921–1-AP, Proteintech), Myc (1:3000, cat# M192-3, MBL), HSP90α (1:3000, cat# 13,171–1-AP, Proteintech), HA (1:3000, cat# M180-3, MBL), Flag (1:5000, cat# F1804, Sigma), FASN (1:1000, cat# 3180, CST), ACC1 (1:1000, cat# F0270, Selleck), p65 (1:1000, cat# 8242, CST), SREBP1 (1:1000, cat# SC-365513, Santa Cruz), Actin (1:1000, cat# F0012, Selleck), Goat Anti-Rabbit IgG (H + L) (1:10,000, cat# 111–035-003, Jackson), Goat Anti-Mouse IgG (H + L) (1:10,000, cat# 115–035-003, Jackson).

Full-length OTUD1 and HSP90α were cloned into pLV-shuttle-EF1α-MCS-FLAG-puro and pHAGE-3Myc-C vectors, respectively. Ubiquitin constructs (K48 and K63) were inserted into pHAGE-3HA-C. sgRNAs targeting OTUD1 were cloned into LentiCRISPR v2.

### Cell culture and transfection

HEK293T, LO2, HepG2, and Huh7 cells were cultured in DMEM supplemented with 10% FBS and 1% penicillin–streptomycin at 37 °C with 5% CO₂, as previously described [48]. Transient transfection was performed with the polyethylenimine (PEI) (Sigma-Aldrich) method for HEK293T cells. Transfection in other cells was performed using PolyJet transfection reagents (SignaGen Laboratories, Rockville, MD, USA).

### BODIPY staining

After treatment of LO2 cells with OA/PA (400/200 µM) for 24 h, the cells were incubated for 30 min with 10 µM BODIPY 493/503 (C2053S, Beyotime) working solution and washed three times with PBS, after which nuclei were stained with DAPI for 5 min. The cells were washed again three times with PBS and imaged by fluorescence microscopy.

### Coimmunoprecipitation and immunoblotting

Co-IP and immunoblotting were performed essentially as described [52]. HEK293T cells were lysed on ice for 30 min in buffer containing 40 mM HEPES (pH 7.4), 120 mM NaCl, 1 mM EDTA, 10 mM sodium pyrophosphate, 10 mM β-glycerophosphate, 0.3% CHAPS, and protease inhibitor cocktail. After centrifugation (12,000 rpm, 15 min, 4 °C), 10% of the supernatant was reserved as input. The remaining lysates were incubated with the indicated primary antibodies overnight at 4 °C, then with 30 µL Protein A/G Plus agarose beads (GenScript) for 2 h. Beads were washed five times with lysis buffer prior to SDS-PAGE. For ubiquitination assays, cells were treated with 10 µM MG132 for 12 h before harvest.

### Immunofluorescence staining

Subcellular colocalization of OTUD1 and HSP90α was examined by immunofluorescence as previously described [52]. LO2 and HepG2 cells grown on coverslips were fixed in 4% paraformaldehyde (10 min), permeabilized with 0.5% Triton X-100 (30 min), and blocked in 3% BSA (2 h, room temperature). Cells were incubated overnight with anti-Flag (1:200, F1804, Sigma) and anti-HA (1:200, AE105, ABclonal) antibodies, followed by Cyanine 3-conjugated anti-mouse IgG (1:100, A22210, Abbkine) and Alexa Fluor 488-conjugated anti-rabbit IgG (1:100, A11008, Invitrogen) for 2 h. Nuclei were counterstained with DAPI, and images were captured on a Zeiss LSM980 confocal microscope.

### RNA isolation and real-time PCR

Total RNA was extracted with TRIzol (Invitrogen) and reverse-transcribed using the 1 st Strand cDNA Synthesis Kit with gDNA wiper (Vazyme, #R312-01) following standard protocols [53]. Quantitative PCR was performed on a QuantReady K9600 system (Life Real) with SYBR Green qPCR Mix (Vazyme, #Q711-02) under the following conditions: 95 °C for 30 s, then 40 cycles of 95 °C for 10 s and 60 °C for 30 s. Relative expression was calculated by the 2⁻^ΔΔCt^ method using β-actin as the internal reference. The following primer sequences were used for qPCR:

- Otud1 (Mouse) qPCR -F: 5′-CAGTATTTGGCTCAGTTGGC-3′
- Otud1 (Mouse) qPCR -R: 5′-CTGTATCTGGGTTTGCTTGC-3′
- Fasn (Mouse) qPCR -F: 5′-AGGTGGTGATAGCCGGTATGT-3′
- Fasn (Mouse) qPCR -R: 5′-TGGGTAATCCATAGAGCCCAG-3′
- Dgat2 (Mouse) qPCR -F: 5′-CTGTGCTCTACTTCACCTGGCT-3′
- Dgat2 (Mouse) qPCR -R: 5′-CTGGATGGGAAAGTAGTCTCGG-3′
- Srebp1c (Mouse) qPCR -F: 5′-GGAGCCATGGATTGCACATT-3′
- Srebp1c (Mouse) qPCR -R: 5′-GGCCCGGGAAGTCACTGT-3′
- Cpt1α (Mouse) qPCR -F: 5′-CTCCGCCTGAGCCATGAAG-3′
- Cpt1α (Mouse) qPCR -R: 5′-CACCAGTGATGATGCCATTCT-3′
- Acc1 (Mouse) qPCR -F: 5′-TGACAGACTGATCGCAGAGAAAG-3′
- Acc1 (Mouse) qPCR -R: 5′-TGGAGAGCCCCACACACA-3′
- Apob (Mouse) qPCR -F: 5′-GCATGAGTATGCCAATGGTCTCC-3′
- Apob (Mouse) qPCR -R: 5′-CTGGTTGCCATCTGAAGCCATG-3′
- Cd36 (Mouse) qPCR -F: 5′-CGACGCGTATGTGGGATGGGGAAGTA-3′
- Cd36 (Mouse) qPCR -R: 5′-GCAAAGGCATTGGCTGGAAGAAC-3′
- IL-1β (Mouse) qPCR -F: 5′-TGGACCTTCCAGGATGAGGACA-3′
- IL-1β (Mouse) qPCR -R: 5′-GTTCATCTCGGAGCCTGTAGTG-3′
- Tnf-α (Mouse) qPCR -F: 5′-GGTGCCTATGTCTCAGCCTCTT-3′
- Tnf-α (Mouse) qPCR -R: 5′-GCCATAGAACTGATGAGAGGGAG-3′
- IL-6 (Mouse) qPCR -F: 5′-TACCACTTCACAAGTCGGAGGC-3′
- IL-6 (Mouse) qPCR -R: 5′-CTGCAAGTGCATCATCGTTGTTC-3′
- Actin (Mouse) qPCR -F: 5′-CATTGCTGACAGGATGCAGAAGG-3′
- Actin (Mouse) qPCR -R: 5′-TGCTGGAAGGTGGACAGTGAGG-3′

### Hepatic triglyceride contents

Liver tissue (30–50 mg) was homogenized in 1 mL cold PBS, and then 4 mL of chloroform/methanol (v/v, 2:1) was added. The mixture was vortexed for 3 min and then kept on ice for 60 min. After a centrifugation at 13,000 rpm for 10 min, the chloroform layer was transferred to a new glass tube with a glass syringe (labeled as Tube 1). Another 3 mL of chloroform/methanol was added into the remaining mixture tube, vortexed, and centrifuged as the first round procedure. The chloroform layer was added into Tube 1 and dried under nitrogen. The triglyceride contents were measured by dissolving lipids with 500–800 µL 1% Triton X-100.

### RNA-seq

Transcriptome sequencing was performed in OA/PA-treated LO2 cells overexpressing OTUD1 and corresponding control cells to identify downstream pathways regulated by OTUD1. High-throughput sequencing was carried out on the DNBSEQ-T7RS platform using a paired-end 150 bp (PE150) sequencing strategy, with a sequencing depth of approximately 6 Gb per sample. Gene expression abundance was calculated and reported as TPM and FPKM values. Differential expression analysis was performed using the DESeq2 package in R based on raw count data. Genes with |log2-fold change|> 1 and false discovery rate (FDR) < 0.05 were defined as differentially expressed genes. Differentially expressed genes were visualized using heatmaps and volcano plots. Gene set enrichment analysis (GSEA) was subsequently performed using pathway datasets obtained from the MSigDB database.

### IP mass spectrometry experiment

IP-MS was performed essentially as previously described [54]. In brief, OTUD1 immunoprecipitates were digested with trypsin, purified on C18 solid-phase extraction columns, and analyzed by LC–MS/MS. Raw data were processed with MaxQuant/Mascot/Proteome Discoverer for peptide identification and quantification.

### Human liver samples

Four MASLD liver tissue samples and their matched adjacent tissues (as controls) were obtained from patients at the time of partial hepatectomy. The clinical characteristics of the four patients are as follows: three females (aged 68, 55, and 54 years) and one male (aged 49 years), all diagnosed with steatohepatitis and hepatocellular carcinoma. Adjacent tissues were histologically confirmed to have no history of viral hepatitis, alcoholic, or drug-induced liver disease. Histological diagnosis was made by qualified pathologists.

### Mice

WT and *Otud1*^*−/−*^ mice on a C57BL/6 J background have been described previously [48]. The mice in the control group were WT littermates. All mice were maintained in specific pathogen-free, humidity- and temperature-controlled microisolator cages with a 12-h light/dark cycle. The sample size was determined from our pilot data. In the pilot study (n = 3 per group), a clear and substantial difference was observed between treatment and control groups. To ensure the reproducibility and statistical robustness of the findings, animals were randomly assigned to experimental groups using a computer-generated random number sequence. Animal inclusion and exclusion criteria were predefined. Only healthy male C57BL/6 J mice were included. Animals meeting preestablished humane endpoints were to be excluded for > 20% unexpected weight loss, severe spontaneous ulceration/infection, or signs of severe distress/immobility. No animals met these exclusion criteria. For each analysis, the exact number of animals (n) was reported for each experimental group. To minimize potential confounders such as treatment order and cage location, the sequence of procedures was randomized, and animals were systematically distributed across cages/racks.

### Diets

Mice were fed either a control diet, a high-fat diet, or a high-fat, methionine-restricted, choline-deficient diet (XTMRCD60). The high-fat diet (XTHF60) provided 60.2% of total calories from fat, 19.7% from carbohydrates, and 20.1% from protein, whereas the control diet (XTCON50J) provided 10.0% of total calories from fat, 69.9% from carbohydrates, and 20.1% from protein. The XTMRCD60 diet was a purified methionine-restricted, choline-deficient diet providing 61.6% of total calories from fat, 20.7% from carbohydrates, and 17.7% from protein.

### Glucose tolerance test (GTT) and insulin tolerance test (ITT)

GTT and ITT were performed on the same cohort of mice with a 2-week interval, with the GTT conducted first, as previously described [55]. For the GTT, overnight-fasted mice received an intraperitoneal injection of glucose (1 g/kg). For the ITT, mice fasted for 6 h were injected intraperitoneally with insulin (1 IU/kg). Blood glucose was measured from the tail vein at the indicated time points.

### Statistical analysis

H&E staining, immunohistochemistry, immunofluorescence, and grayscale analysis of protein bands were all quantified using ImageJ software. Data are presented as the mean ± SD. Statistical analyses were performed using GraphPad Prism version 8.0 (San Diego, CA) (RRID:SCR_002798). Bioinformatics analysis was conducted with R software (version 4.2.1) (http://www.R-project.org/). During mouse allocation, the investigator who generated the random sequence was aware of group assignments. Throughout the conduct of the experiment and outcome assessment, the personnel administering treatments and collecting data were blinded to the group allocations. Data analysis was performed by a statistician who remained blinded to the experimental groups. Statistical significance was evaluated using two-tailed Student's t test, one-way ANOVA, or two-way ANOVA, as appropriate. In the figures, the following notation is used for statistical comparisons between control and experimental groups: *p < 0.05, **p < 0.01, ***p < 0.001, and N.S. for not significant.

## Supplementary Information

Supplementary Material 1.

## Data Availability

All data supporting the findings of this study are available within the article and its Supplementary Information files. Further inquiries can be directed to the corresponding authors. The raw RNA-seq data generated in this study have been submitted to the NCBI database under BioProject ID PRJNA1478745. The IP-MS data have been deposited to the ProteomeXchange Consortium via the iProX partner repository, with the dataset identifier PXD079808.
