# Supplementary material for: Ovarian tumor domain-containing protein 1 deficiency attenuates metabolic dysfunction-associated steatotic liver disease by promoting the ubiquitination of HSP90α in hepatocytes
Source: Mol Biomed. 2026 Sep 20;7:181. doi: 10.1186/s43556-026-00595-1 (PMC13590464; doi:10.1186/s43556-026-00595-1)
Supplement: Supplementary file 1 [file 43556_2026_595_MOESM1_ESM.docx]

**Supplementary Information**

**Ovarian tumor domain-containing protein 1 deficiency attenuates metabolic dysfunction-associated steatotic liver disease by promoting ubiquitination of HSP90α in hepatocytes**

Mei-Qin Huang^1^ ^†^, Si-Yi Wei^1^ ^†^, Meng-Zhe Li^1^, Tian-Yu Chen^1^, Shu-Qin Ou^2^, Meng Gao^1^, Feng Shi^3^, Tao Zhang^4^, Ying-Lan Liu^4^, Meng-Zhuo Qin^1^, Run-Lei Du^1^, Qi Zhang^5*^, Bai-Qi Wang^1,4*^, Xiao-Dong Zhang^1*^.

* Corresponding author: Qi Zhang ([zhangqi@hbut.edu.cn](mailto:zhangqi@hbut.edu.cn)); Bai-Qi Wang (wangbaiqi99@126.com); Xiao-Dong Zhang (zhangxd@usc.edu.cn)

**Supplementary Fig. S1**

(a) Volcano plot of differentially expressed genes in the GSE126848 dataset comparing normal (n = 14) and NASH (n = 16) liver tissues. (b) Boxplots showing OTUD1 expression levels in the GEO dataset GSE126848 (*P*<0.01). (c, d) Representative immunoblots of OTUD1 protein expression in epididymal white adipose tissue (eWAT, c) and brown adipose tissue (BAT, d) from mice fed normal chow (NC) or a high-fat diet (HFD) (n = 4). (e) Representative immunoblots showing OTUD1 protein levels in cells treated with oleic acid/palmitic acid (OA/PA, 0.4/0.2 mM) for 0, 12, or 24 h. GAPDH was used as a loading control (n = 3). (f) NAS scores in livers from NC- and HFD-fed mice (n = 3). (g) NAS scores in livers from normal controls and patients (n = 4). Data are presented as mean ± SD (error bars, SD). *p < 0.05, **p < 0.01, ***p < 0.001; N.S., not significant. Detailed statistical tests are described in the “Statistical analysis” section.

**Supplementary Fig. S2**

(a) Genotyping of wild-type (WT) and *Otud1^-/-^* mice in different tissues by PCR with genomic DNA. (b) Food intake of WT and *Otud1^-/-^* mice fed an HFD for 14 weeks (n = 3). (c) Plasma total cholesterol (TC) (n = 5). (d) NAS scores in livers from WT and *Otud1^-/-^* mice fed an HFD (n = 3). (e) Quantification of Oil Red O (n = 3). (f) Representative images of H&E staining of brown adipose tissue (BAT), epididymal white adipose tissue (eWAT) and inguinal white adipose tissue (iWAT) (n = 3). Scale bars, 50 μm. Data are presented as mean ± SD (error bars, SD). *p < 0.05, **p < 0.01, ***p < 0.001; N.S., not significant. Detailed statistical tests are described in the “Statistical analysis” section.

**Supplementary Fig. S3**

(a-c) Relative mRNA levels of target genes in liver, epididymal white adipose tissue (eWAT), and inguinal white adipose tissue (iWAT) from WT and *Otud1^-/-^* mice (n = 3). (d) Food intake of WT and *Otud1*^-/-^ mice fed an HFMCD for 8 weeks (n = 3). (e) Body weight of WT and *Otud1^-/-^* mice fed an HFMCD for 8 weeks (n = 6). (f) Plasma total cholesterol (TC) (n = 5). Data are presented as mean ± SD (error bars, SD). *p < 0.05, **p < 0.01, ***p < 0.001; N.S., not significant. Detailed statistical tests are described in the “Statistical analysis” section.

 **Supplementary Fig. S4**

(a) Representative mass spectrometry analysis of proteins co-immunoprecipitated with OTUD1 in HEK293T cells.

**Supplementary Fig. S5**

(a) Ubiquitination analysis of wild-type (WT) HSP90α and the K283R and K292R mutants in cells expressing OTUD1, with or without MG132 treatment. (b) Ubiquitination analysis of HSP90α lysine mutants (K407R, K546R, K558R, and K615R) in the presence or absence of OTUD1 and MG132. (c) Coimmunoprecipitation and ubiquitination assay showing the effect of OTUD1 on STUB1-mediated ubiquitination of HSP90α in the presence or absence of MG132.

 **Supplementary Fig. S6**

(a) Immunoblot analysis confirming the stable overexpression of Flag-*OTUD1* in cells, with vector-transduced cells as the control. (b) Immunoblot analysis of *OTUD1* expression in control and *OTUD1*-knockout cells. (c) Viability of HepG2 cells treated with increasing concentrations of PA for 24 h (n = 6). (d–f) Cell viability of HepG2, LO2, and Huh7 cells after treatment with the indicated concentrations of OA/PA for 24 h (n = 6). (g, h) Representative Oil Red O staining images of HepG2 and LO2 cells treated with OA/PA for 24 h (n = 3). Scale bars, 100 μm. (i, j) Quantification of Oil Red O staining intensity in HepG2 and LO2 cells after OA/PA treatment for 24 h (n = 3). (k) Confocal images of LO2 cells transfected with Flag-*OTUD1*; LDs were highlighted with BODIPY 493/503. inset 2 in A depicts an untransfected cell, whereas inset 1 depicts a cell expressing Flag-*OTUD1* (n = 3). Scale bar, 5 μm. Data are presented as mean ± SD (error bars, SD). *p < 0.05, **p < 0.01, ***p < 0.001; N.S., not significant. Detailed statistical tests are described in the “Statistical analysis” section.

 **Supplementary Fig. S7**

(a, b) Immunofluorescence staining of CD206 in liver from WT and *Otud1*^-/-^ mice fed an HFD (a) or HFMCD (b) (n = 3). Scale bar, 40 μm. (c, d) Flow cytometry analysis of CD86 expression (c) and quantification of CD86 mean fluorescence intensity (d) in liver cells from WT and *Otud1*^-/-^ mice under HFMCD (n = 4). (e) Flow cytometry histogram (left) and MFI quantification (right) of CD86 in bone marrow-derived macrophages (BMDMs) treated with LPS for 24h from WT and *Otud1*^-/-^ mice (n = 3). (f) qRT-PCR analysis of relative mRNA levels of pro-inflammatory cytokines (IL-6, IL-1β, TNF-α) in BMDMs from WT and *Otud1*^-/-^ mice (n = 3). Data are presented as mean ± SD (error bars, SD). *p < 0.05, **p < 0.01, ***p < 0.001; N.S., not significant. Detailed statistical tests are described in the “Statistical analysis” section.

 **Supplementary Fig. S8**

(a) Food intake of mice in the AAV-Control and AAV-sh*Otud1* groups during the experimental period (n = 3). (b) Body weight changes in mice from the AAV-Control and AAV-sh*Otud1* groups during the experimental period (n = 6). (c) Serum levels of IL-6 and TNF-α in mice from the indicated groups (n = 5). (d) Representative immunofluorescence images of F4/80 staining in liver sections from AAV-Control and AAV-sh*Otud1* mice. Nuclei were counterstained with DAPI (n = 3). Scale bars, 50 μm. (e) Immunoblot analysis of the indicated proteins in liver tissues from AAV-Control and AAV-sh*Otud1* mice (n = 4). Data are presented as mean ± SD (error bars, SD). *p < 0.05, **p < 0.01, ***p < 0.001; N.S., not significant. Detailed statistical tests are described in the “Statistical analysis” section.
